# ‘A role model is like a mosaic’: reimagining URiM students’ role models in medical school

**DOI:** 10.1186/s12909-023-04394-y

**Published:** 2023-06-02

**Authors:** Isabella Spaans, Renske de Kleijn, Conny Seeleman, Gönül Dilaver

**Affiliations:** Educational Center, University Medical Center Utrecht, Postbus 85500 (HP: HB 4.05), Utrecht, 3508 GA The Netherlands

**Keywords:** Role models, Inclusive medical education, URiM students, Diversity, Inclusion, Concept-guided analysis

## Abstract

**Background:**

Role modelling is a widely acknowledged element of medical education and it is associated with a range of beneficial outcomes for medical students, such as contributing to professional identity development and a sense of belonging. However, for students who are racially and ethnically underrepresented in medicine (URiM), identification with clinical role models may not be self-evident, as they have no shared ethnic background as a basis for social comparison. This study aims to learn more about the role models of URiM students during medical school and about the added value of representative role models.

**Methods:**

In this qualitative study we used a concept-guided approach to explore URiM alumni’s experiences with role models during medical school. We conducted semi-structured interviews with ten URiM alumni about their perception of role models, who their own role models were during medical school and why they considered these figures as role models. Sensitizing concepts guided the topic list, interview questions and finally served as deductive codes in the first round of coding.

**Results:**

The participants needed time to think about what a role model is and who their own role models are. Having role models was not self-evident as they had never thought about it before, and participants appeared hesitant and uncomfortable discussing representative role models. Eventually, all participants identified not one, but multiple people as their role model. These role models served different functions: role models from outside medical school, such as parents, motivated them to work hard. Clinical role models were fewer and functioned primarily as examples of professional behaviour. The participants experienced a lack of representation rather than a lack of role models.

**Conclusions:**

This study presents us with three ways to reimagine role models in medical education. First, as *culturally embedded*: having a role model is not as self-evident as it appears in existing role model literature, which is largely based on research conducted in the U.S. Second, as *cognitive constructs*: the participants engaged in selective imitation, where they did not have one archetypical clinical role model, but rather approach role models as a mosaic of elements from different people. Third, role models carry not only a behavioural but also a *symbolical value*, the latter of which is particularly important for URiM students because it relies heavier on social comparison.

## Background

Dutch medical schools have an increasingly ethnically diverse student population [1, 2], but students who are underrepresented in medicine (URiM) receive lower clinical grades than the ethnic majority group [1, 3, 4]. Furthermore, URiM students are less likely to advance into a medical specialty (known as medicine’s *leaky pipeline* [5, 6]), and they experience unsafety and exclusion [1, 3]. These patterns are not exclusively Dutch: literature reports URiM students dealing with similar challenges in other parts of Europe [7, 8], as well as in Australia and in the U.S. [9–14].

Medical education literature suggests several interventions to support URiM students, one of which is ‘visible ethnic minority role models’ [15]. For medical students in general, exposure to role models is associated with developing their professional identity [16, 17], an academic sense of belonging [18, 19], permeating the hidden curriculum [20] and their choice of clinical field for residency training [21–24]. Amongst URiM students in particular, a lack of role models is commonly identified as a challenge or perceived barrier for achieving study success [15, 23, 25, 26].

Given the challenges of URiM students and the potential value of role models to overcome (parts of) these challenges, this study aims to provide insight into URiM students’ experiences and considerations surrounding role models in medical school. In doing so, we aim to learn more about the role models of URiM students and the added value of representative role models.

### Role models in medical education

Role modelling is considered an important teaching strategy in medical education [27–29]. Role models are amongst the most powerful factors ‘that influence […] a physician’s professional identity’ and as such are ‘fundamental to socialization’ [16]. They provide ‘a source of learning, motivation, self-definition and career guidance’ [30], and they facilitate the acquisition of tacit knowledge and ‘the move from the periphery towards the center of the community’ that students and residents wish to join [16]. If racially and ethnically underrepresented medical students are less likely to identify role models during medical school, this might hinder their professional identity development.

The majority of the research on clinical role models consists of surveys inventorying attributes of excellent clinical teachers, implying that the more boxes a doctor checks, the more likely they are to serve as a role model for medical students [31–34]. This has resulted in a predominantly descriptive body of knowledge about clinical teachers as behavioural models who demonstrate skills that are learnt through observation, leaving room for knowledge about how medical students identify with their role models and why role models are important.

### Defining role models

The importance of role models for medical students’ professional development is widely acknowledged amongst medical education scholars. Discovering more about the processes behind role models is complicated by a lack of definitional consensus and the inconsistent use of research designs [35, 36], outcome variables, methodologies, and contexts [31, 37, 38]. However, there is a consensus that the two main theoretical elements to understand role model processes are *social learning* and *role identification* [30]*.* The first – social learning – derives from Bandura’s theory that people learn via observation and modelling [36]. The second – role identification – refers to ‘the notion that individuals are attracted to people whom they perceive some similarity to’ [30]. 

Major strides in describing role model processes have been made in the field of career development. Donald Gibson disentangled *role models* from the closely related, often interchangeably used terms *behavioural model* and *mentor*, by assigning each of them different developmental targets [30]. Whereas the focus of behavioural models lies in observation and learning, and mentors are characterized by involvement and interaction, role models inspire through identification and social comparison. In this paper, we choose to work with (and thus build on) Gibson’s definition of role models: ‘a cognitive construction based on the attributes of people in social roles an individual perceives to be similar to himself or herself to some extent and desires to increase perceived similarity by emulating those attributes’ [30]. This definition emphasises the weight of social identification and perceived similarity, which are two potential stumbling blocks for URiM students looking for role models.

### Role models for URiM students

URiM students may be disadvantaged by definition: because they are in the minority, they have fewer people who are ‘similar to themselves’ compared to ethnic majority students, and therefore they may have fewer potential role models. As a result, ‘minority youth may often have role models who are not related to their career goals’ [39]. Several studies suggest that demographic similarity (sharing a social identity, for example ethnicity) may be even more important for URiM students than it is for majority students. The added value of representative role models first shows as URiM students consider applying to medical school: social comparison with representative role models provides them with the confidence that ‘someone from their background’ could be successful [40]. In general, minority students with at least one representative role model show ‘significantly greater academic performance’ than students with no role models or only out-group role models [41]. And while majority students in science, technology, engineering, and mathematics gain motivation from both minority and majority role models, minority students risk being *demotivated* by majority role models [42]. The lack of perceived similarity between minority students and out-group role models means they cannot provide ‘concrete information to young people regarding what is possible for them as members of specific social groups’ [41].

The research question in this study is: who were the role models of URiM alumni during medical school? We divide this question into the following sub questions:

1. What is the participants’ definition of a role model?
2. Who were their role models during medical school?
3. Why were these people their role models?

## Methods

### Methodology and research design

**Fig. 1:**
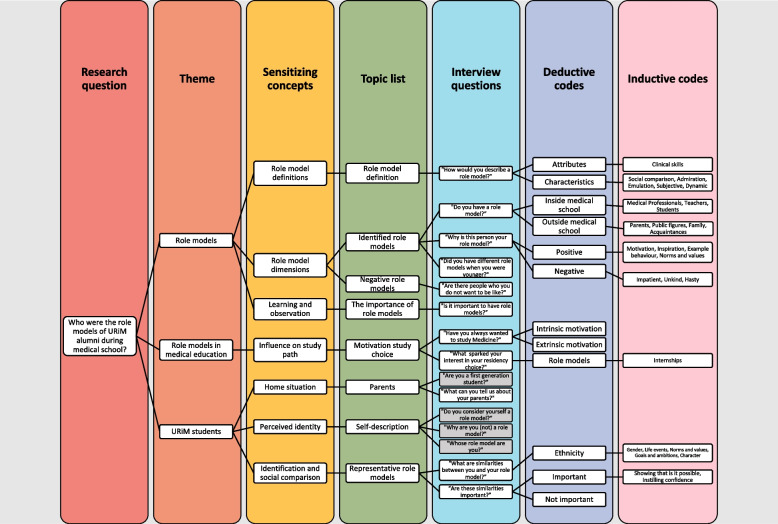
Concept-guided research design

We chose to conduct a qualitative study to facilitate the explorative nature of our research aim, which is to learn more about who the role models of the URiM alumni were and moreover why these people functioned as role models. Our concept-guided approach [43] started by formulating sensitizing concepts which make visible the prior knowledge and conceptual frameworks that influence the researchers’ perception [44]. Following Doorewaard [45] the sensitizing concepts then guided the topic list, the questions of the semi-structured interviews and finally served as deductive codes in the first round of coding. Deviating from Doorewaard’s rigid deductive analysis, we then entered an iterative analysis phase where we complemented deductive codes with inductive data codes (see Fig. 1* Concept-guided research design*).

### Setting

This study was carried out among URiM alumni from the University Medical Center Utrecht (UMC Utrecht) in the Netherlands. Currently, an estimate of under 20% of all medical students at UMC Utrecht have a non-Western migration background.

### Participants

We defined URiM alumni as alumni from the main historically underrepresented ethnic groups in the Netherlands. While acknowledging their diverse ethnic backgrounds, ‘being ethnically underrepresented in medical school’ remained their common denominator.

We interviewed alumni instead of students because alumni can offer a retrospective view that enables them to reflect on their experiences during medical school and because they can speak freely as they are no longer in training. We also wanted to avoid being over-demanding of the URiM students at our institution in terms of participating in studies about URiM students. Experience taught us that conversations where URiM students are approached as such can be very sensitive. Therefore, we prioritized a safe and private setting of one-on-one interviews in which participants could speak freely over data triangulation through other methods like focus groups.

The sample had an even representation of male and female participants from the main historically underrepresented ethnic groups in the Netherlands. At the time of the interviews, all participants graduated medical school between 1 and 15 years ago and were now either in residency or working as a medical specialist.

### Data collection

Through purposive and snowball sampling, the first author approached fifteen URiM alumni who they had no prior relation with from UMC Utrecht via email, ten of which agreed to be interviewed. It was not easy to find alumni from an already very small community who were willing to partake in this study. Five alumni indicated that they did not want to be interviewed as an ethnic minority. The first author conducted one-on-one interviews at UMC Utrecht or in the alumni’s workplace. The topic list (See Fig. 1: Concept-guided research design) structured the interviews, while leaving room for participants to bring up new topics and ask questions. The interviews lasted on average around sixty minutes.

After the first interview where we asked about the participant’s role models quite early on, we noticed that having and talking about representative role models was not self-evident and more sensitive than we anticipated. To establish rapport (‘an essential component of the interview’ involving ‘trust and a respect for the interviewee and the information he or she shares’) [46], we added the topic ‘Self-description’ to the beginning of the interview. This allowed for some small talk and a relaxed atmosphere between the interviewer and the interviewee before we moved on to the more sensitive topics.

We completed the data collection after ten interviews. The explorative nature of this study made it difficult to pinpoint an exact point of data saturation. However, in part thanks to the topic list, the author who conducted the interviews clearly recognized recurring answers early on. After discussing the first eight interviews with the third and fourth author, it was decided to conduct two more interviews, which did not result in new insights. We used audio recordings to transcribe the interviews verbatim—the transcripts were not returned to the participants.

### Data analysis

Participants were assigned codenames (R1 through R10) to pseudonymize the data. The transcripts were analyzed in three rounds:

1. First, we organized the data by interview topic, which was straightforward because the sensitizing concepts, interview topics and interview questions all aligned. This resulted in eight sections with each participants’ comments regarding that topic.
2. Then we coded the data with the deductive codes. Data that did not fit the deductive codes were ascribed inductive codes and labelled as identified themes [47] in an iterative process where the first author discussed the progress with the third and fourth author on a weekly basis, over the course of several months. In these meetings the authors discussed field notes and ambiguous coding cases and deliberated over fitting inductive codes. This resulted in three identified themes: student life and moving out, bi-cultural identity, and a lack of ethnic diversity in medical school.
3. Finally, we summarized the coded sections, added quotes, and arranged them by topic. This resulted in an elaborate overview that allowed us to look for patterns to answer our sub-questions: what is the participants’ definition of a role model, who were their role models during medical school, and why were these people their role models? Participants did not give feedback on the findings.

## Results

We interviewed ten URiM alumni from a Dutch medical school to learn more about their role models during medical school. The results of our analysis are presented below in three themes (role model definitions, identified role models and role model functions).

### Role model definitions

In the definitions of role models, the three most recurring elements were: social comparison (the process of finding similarities between a person and their role model), admiration (looking up to someone) and emulation (wanting to copy or obtain certain behaviours or skills). Below is a quote that contains the elements admiration and emulation.

“The person you look up to, the one that makes you think that’s who I want to be like.” [R4].

Second, we found that all participants described the subjective and dynamic aspects of role models. These aspects describe how people do not have one fixed role model, but how different people have different role models at different times. Below is a quote in which a participant describes how role models change along with a person’s own personal development.

“When your own norms and values change, you can also get another role model, I suppose.” [R7].

### Identified role models

#### Hesitance towards role models

None of the alumni could come up with a role model right away. In analysing answers to the question ‘Who is your role model’, we found three reasons why they found naming a role model difficult. The first reason, that most of them gave, was they had never thought about who their role model was.

“Before this interview I had never thought in terms of role models.” [R9].

The second reason that participants gave was that the word ‘role model’ did not fit their perception of the people around them. Several alumni explained how role model is too grand a label to give to anyone, because nobody is perfect.

“I think it’s something very American, that it’s greater, like ‘That’s the guy I want to be. I want to be Bill Gates, I want to be Steve Jobs’. […] So, I don’t really have one role model in a bombastic way like that, honestly.” [R3].

“I remember several people from my internships that I wanted to be like, but that’s not like saying: this is a role model.” [R7].

The third reason was that participants described having role models as a subconscious process, as opposed to an intentional or deliberate choice that they could easily reflect on.

“I think it’s something you deal with subconsciously. It’s not like saying: ‘that’s my role model, that’s the way I want to be’, but I do think that subconsciously you’re affected by others who have been successful.” [R3].

The participants were noticeably more comfortable discussing negative role models than they were discussing positive role models, sharing examples of doctors they absolutely did not want to be like.

#### Identified role models

**Fig. 2:**
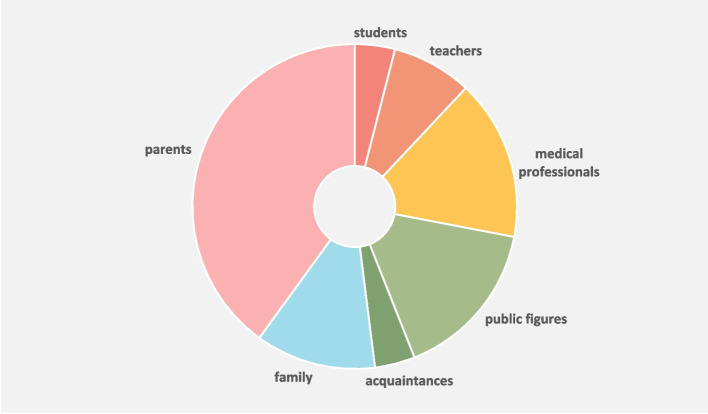
Role models of URiM alumni during medical school

After this initial hesitation, the alumni all identified several people who, in retrospect, may have served as role models during medical school. We grouped these into seven categories, as shown in Fig. 2* Role models of URiM alumni during medical school.*

The majority of the identified role models consisted of people from the alumni’s personal lives. To clearly distinguish these from their role models in medical school, we henceforward split the role models into two categories: role models from inside medical school (students, teachers, and medical professionals) and outside medical school (public figures, acquaintances, family, and parents).

In all cases, what attracted alumni to their role models was the fact that they formed a reflection of the alumni’s own goals, ambitions, norms and values. For example, a medical student who highly valued taking time for patients named a doctor as their role model because they had witnessed them taking time for their patients.

#### Role models as a mosaic

Analysing the alumni’s role models showed that they did not have one all-encompassing role model. Instead, they combined elements of different people to create their own unique, fantasy-like role model. Some alumni just implied this by naming multiple people as role models, but a few of them explicitly described it, as illustrated by the quotes below.

“I think that in the end your role model is like a mosaic of the different people you meet.” [R8].

“I think that in every course, during every internship, I met someone that made me go you’re so good at what you do, you’re such a great doctor, or you’re such a great person, or I’d really like to be like you, or you’re handling that physical exam so well, so I can’t really name one person." [R6].

“It’s not like having one major role model whose name you’ll never forget, it’s more like: you’ve met so many doctors, you compose a kind of average role model for yourself.” [R3].

#### Representative role models

The participants acknowledged the importance of similarity between themselves and their role models. Below is an example of a participant who agreed that a certain degree of similarity is a substantial part of what makes a role model.

“You’re more likely to take someone’s word if they have a similar background as you.” [R3].

We found several examples of similarities that alumni deemed useful, such as similarity in gender, life events, norms and values, goals and ambitions, and character.

“You don’t have to be similar to your role model in terms of appearance, but you do have to have similar characters.” [R2].

“I think that it is important to have the same gender as your role model – women have a bigger impact on me than men.” [R10].

Alumni themselves did not bring up shared ethnicity as a form of similarity. Upon being asked about the added value of having a shared ethnicity, participants answered reluctantly and circuitous. They emphasized that there are more important grounds for identification and social comparison than shared ethnicity.

“I guess that on a subconscious level it would’ve helped if there had been someone with a similar background, ‘like attracts like’. If you have the same background, you have more in common, and you might be more prone to take somebody’s word or be more enthusiastic. But I don’t think this should make all the difference, it should be about what you want to achieve in life.” [R3].

Some participants described the added value of having the same ethnicity as their role model as ‘showing that it is possible’ or as ‘instilling confidence’:

“It may make a difference if they are non-western compared to western, because it shows that it is possible.” [R10].

“For a while it did feel like I needed Moroccan role models. But looking back I don’t think that I needed to see these role models, because I already believed that it existed. […] But having more similarities does kind of give you that extra confidence, like: this is not an illusion that I have. And that things will be okay in the end.” [R8].

### Role model functions

In analysing the responses to ‘Why was this person your role model?’, we found that different role models qualify as such for different reasons: either exhibiting professional behaviour or serving as a source of motivation. Interestingly, these ‘reasons to be considered a role model’ were consistently attributed to either role models inside or outside medical school.

“A role model can have different purposes: you can aspire after a certain position, but also after personality traits like work hard or being nice to someone.” [R10].

#### Role models inside medical school

The function that role models inside of medical school fulfilled for URiM alumni can be summarized as exhibiting professional behaviour. The examples were manyfold and included demonstrating clinical skills, having great teaching skills, interacting with students, and maintaining a healthy work-private life balance. Below is an example of an alumnus whose role model was a surgeon who displayed example behaviour and who they admired.

“… that man, he was just kind of like a… a real personality whenever he entered a room, and also very funny. But most of all, and that’s something I hadn’t seen before him or since him, that he really took students by the hand.” [R7].

#### Role models outside medical school

Role models from outside medical school were considered as such predominantly because of shared norms and values, and to a lesser extent due to specific career paths or professional accomplishments. The majority of URiM alumni did not have role models in their personal life who made them pursue a medical career.

Parents were a source of inspiration and motivation to work hard in medical school for two main reasons. First, parents’ immigration history was often named as a source of inspiration to work hard and persevere. As immigrants, be it migrant workers or refugees, they fled their home country and overcame adversity to offer their children a better future.

“My role model is my mother. She is such a strong woman, she’s been through a lot, fled her country with two young children, a new country, a new language, a new culture, new norms and values, she studied, she raised us, and she is still here for us day and night. She inspires me to always try my very best.” [R6].

Second, parents’ socio-economic status sometimes forced URiM alumni to study hard, because additional tutoring was not financially an option.

“It must have been hard on my parents too, because financially… I remember us having a lot of discussions about the costs of private tutoring. So, it was like: just start by doing your homework properly and then we’ll see.” [R8].

### Identified Theme: Lack of Ethnic Diversity in Medical School

The URiM alumni spontaneously brought up similar topics, resulting in three identified themes: student life and moving out, bi-cultural identity, and a lack of ethnic diversity in medical school. These all alluded to how URiM alumni experienced being a minority in medical school and how having more representation could have changed their experience of being ‘different’. Below we therefore focus on their experienced lack of ethnic diversity in medical school. This lack of ethnic diversity among fellow students and teaching staff made them feel uncomfortable, socially excluded, or hindered their professional development. Most of these experiences occurred during workplace learning.

“I definitely think it would have helped it there would have been more doctors with a minority background.” [R6].

Several participants shared how they were not accepted into certain highly regarded residency training programs, such as general surgery. One extreme example of this was an older participant who was denied a residency position, based explicitly on his migration background.

“He sat next to me and said: ‘Son, I’ll be honest with you: I could mess with you and hire you temporarily, but you won’t make it. You won’t get in. Not because you’re too old, but because you’re a foreigner. You should go and do something else.’” [R4].

Other examples of hinder attributed to a being an ethnic minority in medical school included people making assumptions about participants’ native language, ethnicity, or religion, for example being asked to interpret for people from a different country than their own. Despite introducing this topic, most participants appeared remarkably unfazed about the incidents and assumed that it would be just a matter of time before medical schools will be more ethnically diverse. The participant in the quote below noticed a shift in attitude towards URiM students:

“I think the medical profession and medical education are swiftly changing […] in favor of students with underrepresented backgrounds. […] having a different cultural background is starting to become a positive asset. I’m noticing this in job interviews, like we’re seeing that patients are comfortable around you because of how you look. […] In five years, I think medical education will be drastically different.” [R8].

## Discussion

URiM students receive lower clinical grades than the ethnic majority group, are less likely to advance into a medical specialty, and they experience unsafety and social exclusion. Literature suggests they need more ethnic role models but offers feeble empirical or theoretical underpinning for this solution. We interviewed ten successful URiM alumni to learn about their role models during medical school. We focused on role models encountered during medical school to strengthen the link between our study and medical educators’ everyday practice and what may be within their sphere of influence. Below we will discuss our findings in three themes: having role models is not self-evident, role models as cognitive constructs instead of actual people, and the behavioral and symbolical value of role models.

### Having a role model is not self-evident and can be uncomfortable for Dutch URiM students

When the alumni were asked to give a general description of role models, they noted how role models differ per person, per life phase and for different parts of their lives, thus emphasizing role models’ subjective and dynamic nature. They had difficulty discussing their own role models and needed time to think about whether they even had role models. Finally, they were reluctant to discuss role model ethnicity.

These findings concur with a previous study that compared the role models of Dutch students to those of American students [48]. Like them, we also found that our Dutch participants need a lot of time to think about who their role models are, that they mostly consider their parents as their role models, and that they often do not have role models. The observed hesitancy to speak about role models has also previously been described among Dutch students [48, 49]. In sociology this has been ascribed to the Dutch child-rearing ideal of uniqueness, which for Dutch students is at odds with the concept of aspiring to be like somebody else (whereas these two can ‘coexist without conflict’ among U.S. students) [48, 50]. Consequently, it makes sense that the participants were more comfortable discussing negative role models. This principle also appears to apply to our sample, which consisted mostly of alumni who grew up in migrant households in the Netherlands. Although they were taught their family’s own cultural norms and values, they also grew up within the Dutch society and the Dutch educational system, making it likely that they internalized this typical Dutch take on role models.

The trouble that our participants had to come up with role models contrasts the implicit assumption in most literature that all students have clinical role models [32]. To our knowledge, the struggle that URiM alumni experience with talking about role models has not been acknowledged before in medical education research. Eventually the participants did identify role models, but very few within medical school and even less of those were representative role models. The consequences of the lack of representative role models for the alumni’s professional identity development were difficult for them to pinpoint; possibly because role modelling partly takes place on a subconscious level [51]. As Cruess [16] states: “The learner is generally unaware that she or he is developing a professional identity through this process”. The participants however clearly verbalized how they would have appreciated feeling more represented instead of feeling like, and being treated as, ‘the other’.

The practical consequence of this hesitancy towards role models lies in medical schools’ diversity policies, where role models tend to take up a very central space. Policy makers in Dutch medical schools should acknowledge that the concept of role models is not perceived unequivocally positive and can even be met with students’ resistance. Additionally, we should acknowledge the sensitivity around representative role models for URiM students as this puts the emphasis on their ethnicity, while they often work very hard just to break away from that stigma and do not wish to be addressed as an ethnic minority. A theoretical consequence of this finding is that findings from U.S.-based research on role models in medical education cannot be blindly applied to medical schools outside the U.S., because having role models and talking about them appears to be culturally embedded.

### Role models as cognitive constructs instead of actual people

Our alumni did not identify one all-encompassing role model for themselves in medical school but rather mentioned multiple people who all partially functioned as somewhat of an example figure, both from inside and outside of medical school. Some participants described how they subconsciously composed a fantasy role model out of components of different people in their lives – much like a mosaic.

This approach to role models as a cognitive construct rather than an actual person has been described in organisational behaviour and career management literature, but to the best of our knowledge not yet in the context of medical education. In the field of organisational behaviour and career development it has been described as selective imitation, [52] the composite role model, the cognitive construal of role models, [30, 53] and cherry-picking (‘where rather than mimic the entirety of the role model, the individual just […] attempts to copy the desirable aspects’) [54]. Actively, but not necessarily consciously, selecting specific attributes from various real people (referred to as *proto-models* [55]) and combining them into an imaginary role model is considered ‘a more accurate portrayal’ of how people use role models in everyday life [30].

Career management literature suggests that women as a minority in the workspace carry a heavier cognitive load than men to gather enough proto-models to compose a proper role model because they encounter fewer people who look like them [56, 57]. Our study suggests that the same might apply to URiM students as a minority in medical school.

This approach to role models is quite the opposite of the archetypical view of role models in medical education as an actual doctor who is expected to function as a role model for many students based on possessing a particular set of traits. Viewing role models as a reflection of students’ learning needs also puts into question the manufacturability with which role models are regularly presented (e.g., how to become a role model, how can we use role models to attract students, lists of role model attributes, teaching doctors to behave like role models).

On a policy level, this helps explain the ineffectiveness of so-called ‘diversity hires’: having one minority role model (also known as tokenism [55]) does not suffice, as students need multiple proto-models to compose a role model. Subsequently, more ethnical diversity will also benefit non-URiM students as they will also have more potential proto-models to choose from. Engaging in selective imitation has not been ascribed to minority groups in particular, and further research should include both URiM and non-URiM students to determine to which extent both groups engage in selective imitation.

Further use of career management’s advanced theoretical framework of role models may lead to more novel insights into medical education’s role modelling processes.

### Behavioral and symbolic value of role models

Role models from outside medical school provided alumni primarily with motivation to work hard and role models from inside medical school served mainly as behavioral role models, displaying example behavior as a doctor. Although it makes sense that students do not learn how to be a doctor from role models outside medical school, it is noteworthy that the URiM alumni generally also did not gain motivation or inspiration from role models in medical school. It has been previously noted that URiM students tend to attribute their study success mostly to factors outside the walls of their university, especially to their parents [58].

Research on female role models in the workplace [59] refers to these different types of role model outcomes as the *behavioral* and *symbolic* value of role models. The behavioral value is generally recognized in medical education and focusses on observing and learning from clinical role models to help students become better doctors [34]. The symbolic value however is recognized less often. It relies heavier on the processes of identification and social comparison as the outcomes build on the premise that ‘someone like them’, e.g., someone they share an ethnic background with, can succeed. Symbolic value outcomes include changing students’ cognitive schema of what is possible and other intangible outcomes like hope, inspiration, and a sense of belonging. It also supports the belief that medical school functions as a meritocracy, where all students are equal and judged on their abilities. The participants’ desire to feel more represented aligns with these symbolical role model outcomes. Roughly put, our alumni only had representative role models outside medical school, meaning they were omitted experiencing the symbolic value of role models in medical school. Therefore, medical schools cannot assume that URiM and non-URiM students benefit equally from role modelling in medical school. If URiM students do not have representative role models, they are unlikely to reap their symbolical outcomes.

For medical education and diversity scholars, the symbolical value of role models explains the discrepancy between the ubiquitous presence of clinical role models on the one hand and the recurring call for more role models for URiM students on the other hand. There may be plenty of doctors to provide behavioral value, but not enough representative role models for URiM students to experience their symbolic value. The representative property of clinical role models should be kept in mind when discussing this topic and future research should examine where exactly the added value of representative clinical role models lies.

## Limitations

Below we will present limitations and corresponding suggestions for future research.

First, interviewing alumni meant that although being underrepresented, our participants did not drop out of medical school, are (still) successful, and that they have agreed to be interviewed, unlike other underrepresented alumni that we invited to participate in this study. Future research that includes URiM students who did not finish medical school might result in a different perspective on the importance of role models.

Second, because we only spoke with URiM alumni, we were unable to draw comparisons with the experiences of non-URiM students. Future research should aim to include both URiM and non-URiM students to determine the extent to which results can be ascribed to being underrepresented.

## Conclusions

The findings of this study in which URiM alumni shared their outlook on role models offer three ways to reimagine role models in medical education: 1) as not self-evident and sometimes uncomfortable, 2) as cognitive constructs instead of actual people, and 3) as carrying both a behavioral and a symbolical value. Together, these insights challenge the dominant perception of role models in medical education and underline the importance of creating a learning environment that reflects the racial and ethnical diversity of the students, as it allows all students to identify representative role models to reap both their behavioral and symbolical value. These insights support the added value of representative clinical role models and form the next step in understanding and comprehensively describing the role modelling process.

## Data Availability

The datasets generated and analyzed during the current study are not publicly available due to the protection of participants’ privacy but are available from the corresponding author on reasonable request.

## Abbreviation

URiM: Underrepresented in medicine
